# Editorial: Environmental factors influencing the immune functions during multiple sclerosis

**DOI:** 10.3389/fimmu.2023.1141014

**Published:** 2023-01-20

**Authors:** Joost Smolders, Andrew J. Steelman, Makoto Inoue

**Affiliations:** ^1^ Department of Neurology, MS center ErasMS, Erasmus Medical Center, Rotterdam, Netherlands; ^2^ Department of Immunology, MS center ErasMS, Erasmus Medical Center, Rotterdam, Netherlands; ^3^ Neuroimmunology Research group, Netherlands Institute for Neuroscience, Amsterdam, Netherlands; ^4^ Department of Animal Sciences, College of Agricultural, Consumer, and Environmental Sciences, University of Illinois at Urbana-Champaign, Champaign, IL, United States; ^5^ Neuroscience Program, University of Illinois at Urbana-Champaign, Urbana, IL, United States; ^6^ Division of Nutritional Sciences, University of Illinois Urbana-Champaign Urbana, Urbana, IL, United States; ^7^ Department of Comparative Biosciences, College of Veterinary Medicine, University of Illinois at Urbana-Champaign, Champaign, IL, United States; ^8^ Beckman Institute for Advanced Science and Technology, Urbana, IL, United States

**Keywords:** multiple sclerosis, environmental factors, hormones, Epstein - Barr virus, immunology

Multiple sclerosis (MS) is a neuro-inflammatory and degenerative disease with substantial heterogeneity in presentation, clinical course, and immunopathology. In addition to genetic background, several environmental risk factors are critical mediators of the onset and course of MS (1). Disease heterogeneity dictates the therapeutic efficacy of treatment with disease- modifying therapies. Since environmental factors are known to affect the disease trajectory, it is urgent to identify their specific impact on disease heterogeneity as well as their mechanisms of action. Therefore, this Research Topic was focused on the effects of environmental factors on MS and the identification of their mechanisms of action.

Among others, sex, vitamin D status, and body mass index (BMI) determine the environment in which genetic determinants act, which has been addressed in several contributions to this Research Topic. Genome-wide association studies in MS identified a large number of risk-associated single nucleotide polymorphisms (SNP) (2). Additionally, combinations of SNP genotypes can be associated with specific biological traits, and hereby be exploited to investigate associations of these biological traits with specific disease endpoints in case-control designs (3). Vandebergh et al. showed in their Mendelian randomization study, that SNPs associated with higher BMI and increased IL-6 signaling are enriched in MS. The effect of BMI on MS was partly mediated by IL-6 signaling, elaborating further on the well know interaction between these biological factors (4).

Leffler et al. reviewed the importance of the interaction between sex and MS disease mechanisms. They make a case for taking into account sex when investigating the effect of other environmental risk factors on MS, including female-specific characteristics of anti-viral responses. In particular, the effect of sex hormones on immune function and pathology of MS is highlighted. Along this line, Koetzier et al. report on the synergism between corticosteroids, vitamin D, and sex steroids in the control of potentially pathogenic CD4^+^ T cell activation in MS. This group earlier showed Th17.1 cells (IL17^low^IFNγ^high^GMCSF^high^) to be a major contributor to the CNS-homing T cell pool in MS, which show resistance towards suppression by corticosteroids (5). In their current contribution, the authors show that this resistance can be partly overcome *in vitro* by adding 1,25-dihydroxy vitamin D and sex steroids.

Infection with Epstein Barr Virus (EBV) is one of the most consolidated risk factors for developing MS (6), and may even be a prerequisite (7). Márquez et al. report mechanistic studies on the influence of type I interferons on the role of the murine homolog of EBV (murine gammaherpesvirus 68, γHV-68) in the experimental autoimmune encephalomyelitis (EAE) model of neuroinflammation. They extend on their earlier work in which they showed that latent γHV-68 infection results in amplified CNS-infiltration of CD4^+^ and CD8^+^ T cells in EAE (8). The authors now report that in knockout mice for interferon alpha receptor (IFNAR^-/-^), EAE is more severe with a preserved profound infiltration of CD8^+^ T cells in the case of latent γHV-68 infection likewise the wild-type mice. Contrastingly, the adoptive transfer of γHV-68 infected IFNAR^-/-^mice to wild-type recipients did not result in an amplification of CD8^+^ T cell infiltration. These results suggest that type I interferons could be important for the *in situ* interaction of B and CD8^+^ T cells in neuroinflammation, as has been postulated in MS (9). Herewith, the current study of Márquez et al. helps to understand how the interaction between EBV and type I interferons could contribute to the formation of CD4^+^ and CD8^+^ T cell populations that characterize the pathology of MS (10). In addition, Gottlieb et al. report that T cell responsiveness to brain antigens is another factor relevant in this perspective. They studied the proliferative response of peripheral blood mononuclear cells to denatured, organic-extracted brain homogenate with Carboxyfluorescein succinimidyl ester (CFSE)-labelling, and compared the transcriptome and T cell receptor (TCR) clonality of FACS-sorted proliferated vs. non-proliferated T cells. Brain antigen-responsive cells were enriched for mRNA encoding chemokine receptors and cytokines relevant for neuroinflammation, yet TCR sequences did not overlap with EBV, influenza virus, or varicella zoster virus-responsive cells. These findings support the case that EBV-infection may affect a detrimental cellular response to brain antigens (11), which should be the subject of further study.

Altogether, the work submitted to the Research Topic expanded our knowledge of the mechanisms by which environmental risk factors act on immunological determinants of MS outcomes. Herewith, this Research Topic helped to further shape the research agenda to better understand the contribution of environmental risk factors to the heterogeneity of MS.

## Author contributions

All authors listed have made a substantial, direct, and intellectual contribution to the work and approved it for publication.

## References

[1] Amato MP Derfuss T Hemmer B Liblau R Montalban X Soelberg Sørensen P. Environmental modifiable risk factors for multiple sclerosis: Report from the 2016 ECTRIMS focused workshop mult scler. Mult Scler (2018) 24(5):590–603. doi: 10.1177/1352458516686847 28671487

[2] International Multiple Sclerosis Genetics Consortium. Multiple sclerosis genomic map implicates peripheral immune cells and microglia in susceptibility. Science (2019) 365(6460):eaav7188. doi: 10.1126/science.aav7188 31604244 PMC7241648

[3] Vandebergh M Degryse N Dubois B Goris A. Environmental risk factors in multiple sclerosis: Bridging mendelian randomization and observational studies. J Neurol (2022) 269:4565–74. doi: 10.1007/s00415-022-11072-4 35366084

[4] Carr EJ Dooley J Garcia-Perez JE Lagou V Lee JC Wouters C. The cellular composition of the human immune system is shaped by age and cohabitation. Nat Immunol (2016) 17(4):461–8. doi: 10.1038/ni.3371 PMC4890679 26878114

[5] Koetzier SC van Langelaar J Blok KM van den Bosch TPP Wierenga-Wolf AF Melief MJ. Brain-homing CD4+ T cells display glucocorticoid-resistant features in MS. Neurol Neuroimmunol Neuroinflamm (2020) 7(6):e894. doi: 10.1212/NXI.0000000000000894 33037101 PMC7577536

[6] Bjornevik K Cortese M Healy BC Kuhle J Mina MJ Leng Y. Longitudinal analysis reveals high prevalence of Epstein-Barr virus associated with multiple sclerosis. Science (2022) 375(6578):296–301. doi: 10.1126/science.abj8222 35025605

[7] Abrahamyan S Eberspächer B Hoshi MM Aly L Luessi F Groppa S. Complete Epstein-Barr virus seropositivity in a large cohort of patients with early multiple sclerosis. J Neurol Neurosurg Psychiatry (2020) 91(7):681–6. doi: 10.1136/jnnp-2020-322941 PMC7361012 32371533

[8] Casiraghi C Shanina I Cho S Freeman ML Blackman M Horwitz M. Gammaherpesvirus latency accentuates EAE pathogenesis: Relevance to Epstein-Barr virus and multiple sclerosis. PLos Pathog (2012) 8(5):e1002715. doi: 10.1371/journal.ppat.1002715 22615572 PMC3355105

[9] van Langelaar J Rijvers L Smolders J van Luijn MM. B and T Cells Driving Multiple Sclerosis: Identity, Mechanisms and Potential Triggers. Front Immunol (2020) 11:760. 32457742 10.3389/fimmu.2020.00760 PMC7225320

[10] Hsiao CC Engelenburg HJ Jongejan A Zhu J Zhang B Mingueneau M. Osteopontin associates with brain TRM-cell transcriptome and compartmentalization in donors with and without multiple sclerosis. iScience (2023) 26(1):105785. doi: 10.1016/j.isci.2022.105785 36594029 PMC9804143

[11] Soldan SS Lieberman PM. Epstein-Barr Virus and multiple sclerosis. Nat Rev Microbiol (2023) 21(1):51–64. doi: 10.1038/s41579-022-00770-5 35931816 PMC9362539

